# Italian Hospital Teachers’ Perceptions of Technological and Methodological Innovations after the COVID-19 Pandemic

**DOI:** 10.5334/cie.65

**Published:** 2023-04-14

**Authors:** Francesca Maria Dagnino, Vincenza Benigno, Edoardo Dalla Mutta, Chiara Fante

**Affiliations:** 1 Consiglio Nazionale delle Ricerche, IT

**Keywords:** COVID-19, School-in-Hospital, Remote teaching and learning, Technology Enhanced Learning

## Abstract

The COVID-19 pandemic has had a significant long-term impact on education worldwide. In many countries, schools and universities experienced a rapid switch to emergency remote teaching and learning (ERTL), which affected many education systems in the 2020–21 school year. This was true for the Italian educational context as a whole, including School in Hospital (SiHo) services. This study explored how the SiHo functioned in Italy during the 2020–2021 school year. The aim was to explore what, if any, changes the emergency brought about in educational practices and in the adoption of technologies in this specific context, with a particular focus on any differences between school levels. The study was conducted with 252 SiHo teachers using a questionnaire format. The results showed that after the forced adoption of distance modes during the spring 2020, face-to-face teaching returned to be the prevalent mode in the 2020–2021 school year, with some exceptions for upper-secondary school students (covered by ministerial provisions). The teaching approach that SiHO teachers prefer, both for face-to-face and distance lessons, remains frontal instruction, probably given the particular needs of their students. Younger students probably experienced the most significant changes due to the limits imposed on interpersonal contact in hospitals, which prevented group work and play, previously commonly adopted by kindergarten and primary school teachers. In terms of technology integration in educational practice, teachers stated that they had acquired greater competence in the use of a variety of technological resources.

The COVID-19 pandemic imposed significant changes in educational systems worldwide, affecting the school path of 80% of the world’s student population (United Nations Educational, Scientific and Cultural Organisation [UNESCO], 2020). From March 2020, schools in various countries alternated periods of closure with periods in which they were partially closed or fully open, depending on the spread of the virus and specific national policies. It is widely recognized that this situation had a significant impact on students’ learning and well-being and that prompted an unexpectedly massive use of technologies to ensure continuity of education via remote lessons (Organisation for Economic Co-operation and Development [OECD], 2021a, 2021b). The need to shift from a traditional face-to-face educational paradigm to emergency remote teaching and learning (ERTL) represented a challenge for educational systems, schools, teachers, and families (Chifari et al., 2021), and also affected School in Hospital (SiHo) services – the services that provide educational continuity for hospitalized students – in countries where these are available (Benigno et al., 2020; Caggiano et al., 2021; Gajda et al., 2021).

In Italy, through an administrative order called the Decree of the President of the Council of Ministers (DPCM), all schools were closed from March 2020 to the end of the school year in June, and remote education was adopted. In the subsequent 2020–2021 school year, pupils in primary schools attended face-to-face lessons for the whole year, while secondary schools were closed for some periods, during which remote education replaced face-to-face lessons again. These ministerial provisions, as well as changes in hospital organization and operation, had a significant impact on SiHo services and how they functioned. In fact, during the first months of the COVID emergency (spring 2020), hospitals underwent significant changes at the organizational level: wards were rearranged and (non-COVID) admissions were reduced in terms of numbers and duration (Caggiano et al., 2021; Sainati & Biffi, 2020). Further, teachers were prevented from entering hospitals and hospital school sections. Clearly, this caused an abrupt interruption to face-to-face educational activities (Caggiano et al., 2021), which in some cases were replaced with online learning activities carried out by SiHo teachers.

An initial analysis of the changes to SiHo resulting from the pandemic was conducted by the authors in the spring of 2020 through an in-depth online group interview carried out with Italian SiHo teachers (Benigno et al., 2020). The results of the study are briefly described below. In the light of the extension of Ministry regulations, in the school year 2020–2021 and the changes experienced by SiHo operation in the 2019–2020 school year, we decided to carry out a comprehensive research study dedicated to analysing SiHo functioning after the pandemic emergency; that is, during the 2020–2021 school year. The results of this study may also be read in the light of previous research on SiHo services in Italy (Benigno et al., 2017).

## Context of the Study: The School in Hospital Services in Italy

SiHo is a service that guarantees hospitalized students the universal right to an education (Art. 26 of the United Nations [UN] International Human Rights Declaration; UN, 1948), offering them the opportunity to continue to express themselves and develop in a social context and place of learning. SiHo operates differently in different countries, and in many these services are regulated by specific laws. Currently, SiHo is present in most hospital paediatric departments in Italy, as a result of a long process that eventually led to the development of a European Charter of Rights for Children in Hospital (European Association for Children in Hospital [EACH], 2002).

Indeed, SiHo has a long history in Italy. The first classes for primary education were opened in the 1950s, and now SiHo is available to students at all school levels (Benigno et al., 2017). Over the years, the program has undergone major changes from various perspectives, including pedagogy and organization. In recent times, Italy’s Education Ministry has issued a series of SiHo guidelines that covers the entire sector (Ministero dell’Istruzione, dell’Università e della Ricerca [MIUR], 2019).

SiHo is managed in each Italian region by a Central Hub School, which has a range of responsibilities, including coordination and training of teachers, who enrol without having had any previous SiHo specific training. These hub schools also manage formal relations between individual hospitals and students’ mainstream schools. In response to the particular context in which they function, hospital teachers organize their teaching activities around each student’s state of physical health and psychological well-being. Thus, they design individualised learning pathways for each learner in relation to their age, skill level, and length of hospital stay (Benigno et al., 2017).

Over the years, a number of studies have been performed on SiHo services in Italy (Kanizsa & Luciano, 2006; Mantegazza, 2005; Rivoltella & Modenini, 2012). One recent investigation by Benigno et al. (2017) conducted at the national level on a wide sample of SiHo teachers (*N* = 602) has drawn a detailed picture of teachers’ experience, teaching practices, and technology integration. As to teaching practice, given the complexity of this educational context, the majority of primary and secondary school teachers stated that they opted for an individualized approach, mainly based on lectures, whereas kindergarten teachers often proposed more playful activities and group work.

At the time of the above-mentioned study, the teachers’ digital competencies appeared to be limited: more than half of the respondents did not possess a mobile device and stated they were more familiar and confident with applications for personal computers than those for mobile devices. Further, the teachers reported having medium to high competence with standard productivity software like word processors, spreadsheets, and slideshows, and from low to medium competencies in software for videoconferencing or supporting collaboration, as well as learning management systems (LMS). Perceived competencies in the use of different digital tools seemed to be in relation to the frequency of use, suggesting the need for targeted training to broaden teachers’ repertoire.

With the onset of the COVID-19 pandemic, SiHo services were abruptly curtailed, leading teachers to rethink their standard practices given the need to shift to ERTL. This unexpected and sudden circumstance was explored by Benigno et al. (2020) in a group interview with 12 teachers from different SiHo services. In their testimonies, the teachers highlighted the need to rapidly familiarize themselves with technological tools and resources so they could activate ERTL, even when adopted with a small number of students. Teachers continued to manage distance learning activities using the methods hospital teachers commonly adopt, namely, 1:1 teaching, carried out through videoconferencing. Nevertheless, some attempts were made to carry out group activities and run asynchronous activities using resources like digital repositories. The authors attributed the general lack of educational innovation to the demands of dealing with a very critical situation and responding to the new working context, typically from low levels of technological competency.

After this exploratory study, the question remained whether their experiences during COVID-19 have opened up for long-term changes in the SiHo teachers’ practices from a methodological and a technological perspective. To the best of our knowledge, this issue has not been explored to date and, therefore, is a gap in the literature.

## The Present Study

This study explored whether and how SiHo services in Italy were affected by the changes suddenly introduced by the COVID pandemic lockdown in 2019–2020 and by the restrictions in the following 2020–2021 school year. The focus was particularly on the impact these factors may have had on teaching practices and the use of technologies. The data were collected by means of a questionnaire; SiHo teachers were recruited in the autumn of 2021 using a snowball sampling procedure.

The study was performed within the framework of the CLIPSO project, featuring hybrid classes for SiHo (https://www.progetto-clipso.it/). This initiative sought to reduce the isolation of hospitalized children and support their inclusion in the curricular activities of their mainstream schools.

In this paper, we report our findings on a subset of the survey questions; namely, those related to changes that the COVID emergency may have brought to teaching practices and the adoption of technologies in SiHo contexts. Specifically, the Research Questions (RQs) were as follows:

1. What teaching modes, strategies, and technologies were mostly commonly adopted in the 2020–2021 school year following the emergency?
2. Did these approaches and tools a differ according to school level?
3. What perceptions did teachers have after the pandemic about positive and negative changes, as well as potential improvements brought to SiHo?

## Method

### Design

We carried out a questionnaire-based cross-sectional study involving teachers of SiHo Services in Italy. This method allowed us to collect data from a large sample of teachers working in different areas of the country. The questionnaire was developed on the basis of the authors’ previous research in the field – a group interview carried out in the spring of 2020 (Benigno et al., 2020) and the results of a study of SiHo services in Italy conducted by Benigno et al. in 2017 (see Procedure below).

### Participants

The sample of respondents was comprised of 252 hospital teachers, 218 female (87%) and 33 male (13%). In terms of national geographic distribution, 88 were from the north of Italy (35%), 25 (9.8%) from the centre, and 139 are from the south (54.7%) of the country.

As shown in Table 1, most are over 50 years old.

**Table 1:** Age Distribution (Percentages).


	AGED 19–29	AGED 30–39	AGED 40–49	AGED OVER 50

Female	1%	7%	18%	74%

Male	6%	30%	24%	40%


Seventy percent of the participants had a degree, 30% had a high school diploma, 17% taught kindergarten children, 35% taught primary school pupils, and 24% taught in lower- and upper-secondary schools, respectively. Thirty-five percent had at least 10 years of teaching experience, 13% from 6–10 years, 22% from 2–5 years, and 30% had less than two years’ teaching experience.

### Procedure

The questionnaire (see Appendix) was made up of 40 questions. The items consist of a mix of closed [Likert scale and multiple choice] and open-ended questions, organized into six sections:

1. **personal information:** 10 closed and open-ended questions about teachers’ gender, age, educational background, geographical area, experience in teaching, etc.;
2. **teaching organization:** nine closed and open-ended questions about the hospital ward in which teachers taught, their relationships with the healthcare staff, face-to-face/distant teaching during the pandemic, changes in hospital organization affecting teaching, etc.;
3. **methodology:** four closed and open-ended questions about the teaching methods adopted in both face-to-face and distance activities;
4. **technology:** five questions about technologies (hardware and software) employed during the pandemic;
5. **hospitalized students and remote education with mainstream schools:** nine closed and open-ended questions exploring the relationships between the hospitalized students and mainstream schools (formal and informal contacts with peers and teachers), remote education etc.;
6. **reflection on the pandemic’s effects on school in hospital:** three open-ended questions about positive and negative changes, and potentialities of the SiHo service.

After the initial design, the questionnaire was tested with a small group of teachers, then administered online through the open-source software LimeSurvey and spread through the snowball sampling technique between SiHo teachers. Before filling in the questionnaire, teachers were informed about the aim of the research and signed a consent form.

The present discussion presents an analysis of the results from Sections 1, 3, 4, and 6.

### Data Analysis

Statistical procedures (descriptive and inferential) were performed using SPSS (version 26). Not all teachers answered all sections of the questionnaire; statistical analysis was performed using pairwise deletion.

Regarding RQ1, percentages were calculated for the frequency of use of the different teaching modes, teaching strategies, and technologies adopted.

Analysis was performed of the dependent variables regarding teaching strategies used in face-to-face and distance education [RQ1] (Methodology section of the survey), with one-way analysis of variance by ranks (Kruskal-Wallis test) and with school level [RQ2] as a between-subjects independent variable (Kindergarten, Primary School, Lower-Secondary School, and Upper-Secondary School). The same analysis was performed on the dependent variables regarding technologies used in distance education (Technology section of the questionnaire). Alpha was 0.05. Post-hoc comparisons were performed using the Mann-Whitney test corrected with the Bonferroni procedure.

To compare the school levels in face-to-face and distance learning activities [RQ2], a crosstabs analysis and chi-square comparison of the frequency of the two modes was performed. The same analysis was used to examine differences in the changing use of technology and tools in face-to-face education [RQ1]. Alpha was 0.05; adjusted standardized residuals (RSA) were calculated. Effect sizes were calculated (Cohen’s *w* for chi-square and Spearman’s *r* for the other analysis) as well.

For RQ3, analysis of the open-ended questions was carried out using a thematic analysis approach (Braun & Clarke, 2006; Kelle, 2007). Initially, two researchers read all the data independently, identifying the emerging conceptual categories (codes). The codes were then organized into themes embodying similar meanings (Vaismoradi et al., 2016). An inductive, or bottom-up, approach was used; that is, the researchers were not guided in any way by preconceptions or hypotheses, so the process was fully inductive. Themes and codes related to teaching and technology issues were selected, and these are reported in the results section below, under the three questions.

## Results

### Teaching Modes and Strategies

According to questionnaire answers, both face-to-face and distance learning modes were adopted during the 2020–2021 school year [RQ1]; face-to-face mode seemed to be prevalent since 35% of the teachers stated that they taught that way “Always” and 25.5% “Most of the time;” only 10.3% answered “Never” (see Table 2).

**Table 2:** Frequency of Employing the Various Teaching Modes (Percentages).


	NEVER	FOR A FEW MONTHS	FOR ABOUT HALF OF THE SCHOOL YEAR	MOST OF THE TIME	ALWAYS

Face-to-face	10.3%	13.6%	15.6%	25.5%	35%

Distance	41.2%	28.4%	9.9%	11.5%	9.1%


Given that face-to-face is the long-established teaching mode adopted in SiHo services, it can reasonably be assumed that the shift to distance education in this period was a response to the pandemic restrictions.

To identify the relationship between the frequency of the two modes and school levels, a chi-square test was carried out [RQ2]. As to the frequency of face-to-face education at the different school levels, the analysis revealed a significant difference between school levels [χ2 (12) = 30.44; *p* < 0.05]. The effect size was moderate (*w* = 0.35). The analysis of the adjusted standardized residuals showed that for Upper-Secondary School, the actual frequency was significantly higher than the expected frequency for the option “For a few months” (*z* = 3.4; *z*crit. = 2.6) and lower for the option “Always” (*z* = –4.1; *z*crit. = 2.6). This indicates that Upper-Secondary School teachers taught face-to-face less frequently than expected.

With regard to the frequency of distance activities, the analysis also revealed a significant difference between school levels [χ2 (12) = 59.78; *p* < 0.01]. The effect size was moderate (*w* = 0.50). The analysis of the adjusted standardized residuals showed that for Primary School, the actual frequency was significantly higher than the expected frequency for the option “Never” (*z* = 3.8; *z*crit. = 2.6), indicating that distance education was never used in many SiHo settings for primary students. For the Upper-Secondary School, the actual frequency was significantly higher than the expected frequency for the options “Always” (*z* = –5.7; *z*crit. = 2.6) or “Most of the time” (*z* = 4.4; *z*crit. = 2.6), indicating a higher frequency of use of distance learning at this school level. These data also reflect what was happening in mainstream schools.

Among participants who taught in the face-to-face mode, the most common teaching methods adopted [RQ1] were Lectures (48.3% answered “Always” and 33% “Often”), followed by Teaching essential subject area knowledge^1^ (17.2% answered “Always” and 46.3% “Often”), Drill & practice (16.7% answered “Always” and 33% “Often”), and Playful activities (49.3% answered “Always”). The less common methods were Group work (51.2% answered “Never” and 20.7% “Rarely”) and Gamification (31.5% answered “Never” and 17.7% “Rarely”). See Table 3.

**Table 3:** Frequency of Teaching Methods Adopted in Face-to-Face Mode (Percentages).


	NEVER	RARELY	SOMETIMES	OFTEN	ALWAYS

Lectures	4.4%	2%	12.3%	33%	48.3%

Group work	51.2%	20.7%	13.8%	9.9%	4.4%

Brainstorming	26.1%	10.3%	31%	22.2%	10.3%

Drill & practice	21.2%	3.9%	25.1%	33%	16.7%

Project-based learning	23.2%	19.7%	29.6%	22.2%	5.4%

Gamification	31.5%	17.7%	31%	15.3%	4.4%

Playful activities	13.8%	14.8%	22.2%	0%	49.3%

Teaching essential knowledge	6.4%	3.9%	26.1%	46.3%	17.2%

Other strategies	56.4%	5.4%	21.3%	14.9%	2%


A blank area was available for adding other teaching methods not listed in the questionnaire. Here, teachers mentioned storytelling, problem solving, cooperative learning, debate, and some other methods.

Among participants who taught in distance mode, the most widespread methods [RQ1] remained Lectures (73.3% answered “Always”) followed by Teaching essential knowledge (20.6% answered “Always” and 38.9% “Often”) and Drill & practice (14,5% answered “Always” and 33.6% “Often”). Conversely, employment of Playful activities was significantly lower than for face-to-face, indicating difficulty in proposing playful activities in a different mode. The least common methods were Group work (71.8% answered “Never” and 12.2% “Rarely”), Gamification (45.8% answered “Never” and 10.7% “Rarely”), and Project-based learning (37.4% answered “Never” and 16% “Rarely”). See Table 4.

**Table 4:** Frequency of Teaching Methods Adopted in Distance Mode (Percentages).


	NEVER	RARELY	SOMETIMES	OFTEN	ALWAYS

Lectures	8.4%	7.6%	10.7%	0%	73.3%

Group work	71,8%	12.2%	7.6%	0%	8.4%

Brainstorming	25.2%	13%	31.3%	20.6%	9.9%

Drill & practice	20.6%	6.1%	25.2%	33.6%	14.5%

Project-based learning	37.4%	16%	26%	16%	4.6%

Gamification	45.8%	10.7%	26.7%	14.5%	2.3%

Playful activities	33.64%	12.2%	22.9%	24.4%	6.9%

Teaching essential knowledge	7.6%	4.65%	28.2%	38.9%	20.6%


To identify possible differences in teaching methods used in relation to the school level teachers taught [RQ2], a one-way analysis of variance by ranks (Kruskal-Wallis test) was performed with school level as the between-subjects variable (Kindergarten, Primary School, Lower-Secondary School, and Upper-Secondary School). Analysis was performed both for face-to-face and distance modes. Post-hoc comparisons were performed with the Mann-Whitney test corrected with the Bonferroni procedure.

Considering face-to-face teaching methods, analysis revealed a significant difference concerning the use of Drill & practice [χ2 (3) = 31.101; *p* < 0.001], Group work [χ2 (3) = 8.07; *p* < 0.05], Project-based learning [χ2 (3) = 26.75; *p* < 0.001], Gamification [χ2 (3) = 17.91; *p* < 0.001], and Playful activities [χ2 (3) = 71.06; *p* < 0.001].

Post-hoc comparisons revealed:

- less use of Drill & practice in Kindergarten than Primary School (*z* = 5.29, *p* < 0.001, *r* = 0.33), Lower- (*z* = –4.68, *p* < 0.001, *r* = 0.29), and Upper-Secondary School (*z* = –3.25, *p* < 0.001, *r* = 0.20);
- greater use of Group work in Kindergarten than Upper-Secondary School (*z* = 2.67, *p* < 0.05, *r* = 0.17);
- less use of Project-based learning in Upper-Secondary School than Primary School (*z* = 4.07, *p* < 0.001, *r* = 0.25) and Kindergarten (*z* = 3.93, *p* < 0.001, *r* = 0.25);
- greater use of Gamification in Primary School than Upper-Secondary School (*z* = 4.08, *p* < 0.001, *r* = 0.26);
- Playful activities were more common in Kindergarten than Lower-Secondary School (*z* = 4.97, *p* < 0.001, *r* = 0.31) and Upper-Secondary School (*z* = 7.26, *p* < 0.001, *r* = 0.45); analysis also revealed greater use of Playful activities in Primary School than Lower-Secondary School (*z* = 3.81, *p* < 0.001, *r* = 0,24) and Upper-Secondary School (*z* = 4.08, *p* < 0.001, *r* = 0.26); similarly, it was greater in Lower-Secondary School than in Upper-Secondary (*z* = 2.73, *p* < 0.001, *r* = 0.17).

Regarding distance teaching strategies, analysis of variance by ranks revealed a significant difference regarding the use of Drill & practice [χ2 (3) = 14.15; *p* < 0.05], Project-based learning [χ2 (3) = 9.99; *p* < 0.05], Gamification [χ2 (3) = 20.56; *p* < 0.001], Playful activities [χ2 (3) = 38.02; *p* < 0.001], and Lectures [χ2 (3) = 10.14; *p* < 0.05].

Post-hoc comparisons showed:

- less use of Drill & practice in Kindergarten than Primary School (*z* = –3.32, *p* < 0.001, *r* = 0.21) and Lower-Secondary School (*z* = –3.41, *p* < 0.05, *r* = 0.21);
- greater use of Project-based learning in Primary School than Upper-Secondary School (*z* = 3.01, *p* < 0.05, *r* = 0.19);
- greater use of Gamification in Primary School than Upper-Secondary School (*z* = 4.07, *p* < 0.001, *r* = 0.25) and Kindergarten (*z* = –3.13, *p* < 0.05, *r* = 0.20);
- Playful activities were used more commonly in Primary School than in Upper-Secondary School (*z* = 5.27, *p* < 0.001, *r* = 0.33) and in Kindergarten more than in Upper-Secondary School (*z* = 4.57, *p* < 0.001, *r* = 0.29);

No significant difference in the use of Lectures was observable in the post-hoc comparison.

### Technologies

Teachers were asked which technologies they used for carrying out their lessons and if their habits had changed due to the emergence of the pandemic [RQ1]; namely, if their use of digital devices had decreased, increased, or remained stable (see Table 5).

**Table 5:** Changes in Frequency of Use of Different Devices (Percentages).


	DECREASED	STABLE	INCREASED	NO DATA

PC/laptop	2.7%	24.4%	56.4%	16.4%

Tablet	3.6%	31.1%	40.9%	24.4%

Smartphone	0.9%	30.4%	53.1%	15.6%


As illustrated in Table 5, teachers’ use of devices tended to increase, especially for PC/laptop and smartphones, for which more than half of the respondents stated that their use increased.

In order to detect a possible relationship between changes in the use of the different devices and school levels [RQ2], a chi-square test was carried out for each of the use rates. Analysis revealed that the only significant difference among the school levels was for personal computer/laptop use [χ2 (12) = 59.78; *p* < 0.01; *z*crit. = 2.5]; the effect size was high (*w* = 0.52). PC/laptop use increased more frequently among Upper-Secondary School teachers (*z* = 3.5; *z*crit. = 2.5) and less frequently among Primary School teachers (*z* = –2.7; *z*crit. = 2.5).

The teachers were also asked what kind of resources they used for teaching face-to-face during the pandemic [RQ1] (see Table 6). Common productivity software applications (73.1%) and Online educational resources (83.8%) were the most commonly used, followed by Shared online workspaces (66.5%).

**Table 6:** Resources Used for Carrying out Face-to-Face Lessons (Percentages).


	USED	NOT USED

One or more common productivity applications (word processing, spreadsheets, presentations, etc.)	73.1%	26.9%

Shared online workspaces (Google Drive, Dropbox, etc.)	66.5%	33.5%

Collaborative writing applications (Wiki, Google Docs, Book creator, etc.)	43.7%	56.3%

Educational robotics and coding applications (Bee bot, Ozobot, Scratch, etc.)	17.9%	82.1%

Other cloud applications (i.e., Padlet, Kahoot, etc.)	40.4%	59.6%

Online educational resources	83.8%	16.2%


Teachers declared that their technology use changed in response to the pandemic, as shown in Table 7; a tendency towards increased use was evident for online workspaces (49.8%) and educational resources (47.3%).

**Table 7:** Changes in the Use of Resources (Percentages).


	DECREASED	STABLE	INCREASED	NO DATA

One or more common productivity applications (word processing, spreadsheets, presentations, etc.)	5%	35.8%	37.8%	21.4%

Shared online workspaces (Google Drive, Dropbox, etc.)	2%	26.3%	49.8%	21.9%

Collaborative writing applications (Wiki, Google Docs, Book creator, etc.)	1%	32.3%	31.4%	35.3%

Educational robotics and coding applications (Bee bot, Ozobot, Scratch, etc.)	4%	35.7%	7.5%	52.8%

Other cloud applications (i.e., Padlet, Kahoot, etc.)	2.5%	30%	28%	39.5%

Online educational resources	1.5%	35.86%	47.3%	15.4%


Again, we explored the potential relationship between change in the resources used to carry out lessons and school levels through a chi-square test [RQ2]. The analysis shows a difference linked to school levels and the use of common productivity applications [χ2 (12) = 59.78; *p* < 0.01; *z*crit = –2.5]. Analysis of the adjusted standardized residuals showed that for the option Stable the actual frequency at the Primary School level was significantly higher than the expected frequency (*z* = 2.5; *z*crit. = 2.5), while for Lower-Secondary School it was significantly lower than expected (*z* = –3.1; *z*crit. = 2.5). This means that use remained more stable among Primary School teachers and changed among Lower-Secondary School teachers. The effect size was high (*w* = 0.55).

The question about the type of resources was also posed in relation to distance education (see Table 8). Analysis of the use of technologies in distance education at the different school levels revealed significant differences for use of Videoconferencing [χ2 (3) = 45,07; *p* < 0.001], Platforms [χ2 (3) = 9.6; *p* < 0.05], Apps [χ2 (3) = 19.25; *p* < 0.001], Sharing environments [χ2 (3) = 7.9; *p* < 0.05], Writing environments [χ2 (3) = 10.54; *p* < 0.05], and Online resources [χ2 (3) = 8.8; *p* < 0.05].

**Table 8:** Resources Used for Carrying out Distance Lessons (Percentages).


	NEVER	RARELY	SOMETIMES	OFTEN	ALWAYS

Electronic school roll	36.9%	3.1%	1.5%	3.8%	54.6%

Videoconference systems (Skype, Zoom)	12.3%	4.6%	10.8%	15.4%	56.9%

Instant messaging (WhatsApp, Telegram, etc.)	10.8%	3.1%	11.5%	40.8%	33.8%

Platforms for remote education (Moodle, Classroom, etc.)	30.8%	8.5%	19.2%	14.6%	26.9%

Publishers’ applications	53.1%	9.2%	12.3%	16.2%	9.2%

Shared online workspaces (Google Drive, Dropbox, etc.)	27.7%	7.7%	20.8%	26.9%	16.9%

Collaborative writing applications	51.5%	13.8%	13.1%	14.65%	6.97%

Other cloud applications (Padlet, Kahoot, etc.)	54.65%	9.2%	17.78%	13.1%	5.4%

Online educational resources	6.2%	6.9%	32.3%	42.3%	12.3%


Post-hoc comparisons revealed:

- less use of Videoconferencing in Kindergarten than Primary School (*z* = –4.08, *p* < 0.001, *r* = 0.26), Lower-Secondary School (*z* = –3.86, *p* < 0.001, *r* = 0.24), and Upper-Secondary School (*z* = –6.52, *p* < 0.001, *r* = 0.41);
- more frequent use of Videoconferencing in Upper-Secondary School than in Primary School (*z* = –2.97, *p* < 0.05, *r* = 0.19) and Lower-Secondary School (*z* = –3.18, *p* < 0.05, *r* = 0.20);
- less use of Platforms in Kindergarten than Primary School (*z* = –2.75, *p* < 0.05, *r* = 0.17) and Lower-Secondary School (*z* = –2.98, *p* < 0.05, *r* = 0.19);
- less use of Apps in Kindergarten than Lower-Secondary School (*z* = –2.94, *p* < 0.05, *r* = 0.18) and Upper-Secondary School (*z* = –4.1, *p* < 0.001, *r* = 0.26);
- greater use of Apps in Upper-Secondary School than Primary School (*z* = –2.64, *p* < 0.05, *r* = 0.17);
- less use of Writing environments in Kindergarten than Primary School (*z* = –2.94, *p* < 0.05, *r* = 0.18) and Upper-Secondary School (*z* = –3.07, *p* < 0.05, *r* = 0.19).

No significant differences in the use of Sharing environments and Online resources were observable in post-hoc comparison.

### Teaching and Technology: Additional Elements

The last section of the questionnaire presented three open questions investigating the pandemic’s effects on School in Hospital [RQ3]. Specifically, teachers were asked to identify positive and negative issues that emerged, and which aspects of SiHo services needed improvement.

Among the **positive aspects of their experience**, the teachers recognized that they had learned new technological skills and that distance learning can be a resource for hospitalized students.

*“One significant benefit was continuity of individualized learning paths initiated in hospital, which could continue with integrated digital didactic even during periods when the student was in precautionary isolation (periods in which the hospital teachers could not enter the student’s room) and also in brief periods of discharge between hospitalizations” […]*.

In addition, the teachers reported general innovation in teaching related to the use of new teaching methodologies and improved scheduling of educational activities.

“*Increased creativity in the use of different and more innovative teaching tools, especially digital-based ones.”*

Regarding **negative issues**, problems were identified both at methodological and technological levels. For example, teachers reported that hospitals’ pandemic regulations caused serious limitations to their activities, including the inability to implement group activities and to use traditional educational tools (e.g., “*inability to quickly share materials such as pencils and paper*.”).

At the technological level, a number of teachers highlighted internet connection problems:

“*Throughout the hospital* [there were] *considerable connection difficulties, solved only by* [using] *learners’ personal internet accounts*.”

Moreover, the teachers also noted increased difficulties in managing foreign students and some subjects:

*“It was (also) extremely difficult to get through to foreign students. For them, the main trouble was understanding the instructions without body language support.”*

Among the **issues** that teachers identified as **requiring improvement** in the future, they mentioned the need for better technological solutions and technological resources to support distance learning, given that this approach can enhance connections and collaboration with hospitalized students’ mainstream schools.

*“There is certainly a need to increase the multimedia tools available to teachers and* [improve] *Wi-Fi connections in the wards.”*

*“The effectiveness of distance learning could be improved using technology solutions, with the aim of stimulating and involving the pupil, even at a distance.”*

*“Interaction with schools could be increased through distance learning as well.”*

Table 9 shows the main themes that emerged from the qualitative analysis of the questionnaire’s open-ended questions and related codes. A brief description of each code is given, together with an example.

**Table 9:** Themes, Codes, Descriptions, and Examples Emerging from the Questionnaire’s Open-Ended Questions.


	THEMES	CODES	DESCRIPTIONS	EXAMPLES

Q1: Positive Aspects				
	Technologies	Learning new technological skills	Teachers recognizd the positive effect of improving their skills in the use of technology	*“The spread of the pandemic, however, forced us to specialize more in the educational and technological tools available to us.”*

	Teaching innovation	Blended teaching	Teachers recognized the positive effect of blended instruction on hospitalized students’ learning path	*“One significant benefit was continuity of the personal learning paths started in the hospital, which, with integrated digital didactic, could continue throughout the periods of students’ precautionary isolation and also during brief hospital discharges.”*

		Need to plan educational activities	Teachers reported that they had improved their planning of educational activities	*“We learned how to plan our synchronous and asynchronous lessons more efficiently.”*

		Use of new teaching methodologies	Teachers reported that they had tested new teaching methodologies in response to the emergency condition	*“Increased creativity in the use of different and innovative teaching tools, especially digital-based ones.”*

	Distance Learning	Teacher resource	Teachers recognized that distance learning can be a resource in their practice	*“The [forced] discovery of distance learning as a viable resource.”*

		Student resource	Teachers reported that distance learning had improved the social well-being of hospitalized students	*“Distance learning made it possible for hospitalized children to feel like peers who are unable to be present in the classroom.”*

Q2: Negative Aspects				
	Teaching limitations	Inability to perform group activities	Teachers reported that they had not been able to implement group teaching activities	*“Inability to implement group activities with other hospitalized students.”*

		Inability to use some traditional educational tools	Teachers reported that they were unable to use some traditional teaching tools (e.g., Lego, books, games)	*“Inability to quickly share materials such as pencils and paper.”*

	Distance Learning	Internet connection problems	Teachers reported that Internet connection problems in the hospital had been a problem for distance learning	*“Internet connection failures throughout the hospital.”*

		Organizational issues	Teachers reported problems in planning distance education in the hospital school setting	*“Difficulties in organizing distance learning lessons with students admitted for [only] a few days.”*

		Distance learning difficulties related to school subject area	Teachers experienced more difficulties in science subjects than humanities during distance learning lessons	*“Difficulties were more likely to occur in science subjects. In humanities subjects. there were no major problems.”*

		Distance learning difficulties with foreign students	Teachers noted more language difficulties when interacting with foreign students during distance learning	*“It was extremely difficult (also) to get through to foreign students. For them, the main trouble was understanding instructions without body language support.”*

		Distance learning is less stimulating	Teachers reported that distance learning reduced their capacity to convey passion as they used to do during face-to-face activities	*“The side effect of distance learning is, because of the distance, teachers are not able to convey the same passion as they normally do during face-to-face activities. Distance learning is surely less successful in stimulating students’ minds.”*

Q3: Issues to be improved				
	Distance Learning	Improving distance learning implementation	Teachers considered the possibility of improving distance learning teaching with technology solutions	*“Distance learning effectiveness could be improved using technology solutions so as to stimulate and involve the pupil even at a distance.”*

		Distance learning as mediation with schools of origin	Teachers considered distance learning as a solution for increasing connections and mediation with mainstream schools	*“Interaction with schools also could be increased through distance learning.”*

	Technology	Improving technology resources	Teachers expressed the need to increase the technological resources available in hospitals dedicated to educational purposes	*“There is certainly a need to increase the multimedia tools available to teachers and improve Wi-Fi connections in the wards.”*

		Improving hospitals’ Wi-Fi connection	Teachers expressed the need to improve Wi-Fi connection to ensure a good standard of distance learning	*“Internet connection troubles throughout the hospital. The only alternative is using the connection of students’ own devices.”*

		Adopting digital school roll	Teachers expressed the need to enhance the digital school roll system so it is as easy to use as the one adopted in mainstream schools	*“The hospital school roll is a bit intricate; could be more user-friendly, more similar to digital school records adopted in mainstream schools.”*


## Discussion

In the 2020–2021 school year, the SiHo services in Italy were affected by the consequences of the pandemic on multiple levels. In addition to regulations from the Italian Ministry of Education, teachers were affected by several changes and limitations linked to modifications in hospital routines, such as the closure of common spaces and limits on sharing materials.

In relation to teaching modes, the teachers adopted both remote and face-to-face teaching, with the latter being prevalent. Unlike the initial emergency phase, when teachers could not access hospitals, in the subsequent phases they were treated in the same way as health workers, being subjected to a range of medical control routines to reduce the spread of the virus. This treatment indicates considerable sensitivity on the part of the hospital towards young patients’ schooling, recognizing it plays a central role in their care plan.

Although hospital teachers in general were able to give face-to-face lessons, distance learning was mainly used by Upper-Secondary School teachers. This finding is in line with what happened outside the hospital school context, since both mainstream schools and hospital schools followed the ministry regulations, which envisaged remote education for Upper-Secondary students when the virus was most widespread. Upper-Secondary School had less difficulty in applying distance learning, both during the first emergency phase (Gentile et al., 2021) and in subsequent phases. While more senior students also reported difficulties (Chifari et al., 2021; Khlaif et al., 2021), their greater ability to self-regulate and manage technological resources facilitated remote interaction and communication (Bergdahl et al., 2020).

The preferred teaching approach in the context of SiHO, both face-to-face and remote, remains the frontal lesson, confirming findings already published in the literature (Benigno et al., 2017). This dominance of lecture-style teaching probably reflects the prevailingly one-to-one mode adopted for dealing with issues related both to the students’ health status and organizational matters. Furthermore, COVID-19 restrictions made any level of group work impossible to carry out in face-to-face teaching activities. Employment of playful activities decreased in distance mode, probably due to the limited time available for lessons and teachers’ limited familiarity with the opportunities offered by digital technologies in this regard. Hints of general innovation in teaching emerged more frequently from answers to open questions than from closed questions. The likely reason for this is that the teachers probably perceived the transition to remote education itself as a source of innovation in their teaching practice.

It comes naturally to draw a parallel with mainstream school. Despite the significant investments made over the years to integrate ICT into teaching and learning, the poor integration of ICT into teachers’ daily routines is well known (European Commission, 2019). Several variables seem to have contributed to this, in particular, skills in the use of technologies played a decisive role. As reported by the Centro Studi Investimenti Sociali (CENSIS) (2020), digital competence is considered inadequate and insufficient (77.2%); further, “technological enthusiasm” (70.9%) seems to have favoured the use of technologies in schools but with a traditional teaching approach.

In addition, resistance to ICT-related changes, lack of time to adopt ICT in daily classroom activities, and lack of technical staff to support teachers are further variables associated with poor integration of ICT in educational practices (Buabeng-Andoh, 2012; European Commission, 2019). Concerning teaching practices, teachers seemed to have replicated online the type of lecture they used to deliver face-to-face, as reported in other studies (Carretero Gomez et al., 2021), showing limited familiarity with the pedagogical implications for a successful integration of technologies. The lack of pedagogical innovation detected in the SiHo context reflects the trend among mainstream teachers, despite the adoption of training models (see, e.g., Benigno et al., 2018) based on frameworks and models that emphasize the integration of technological, pedagogical, and organizational dimensions (Bocconi et al., 2018; Mishra & Koehler, 2006; Redecker, 2017).

With regard to teaching strategies and the difference in face-to-face teaching activities at different school levels, game-based strategies were much more commonly used at the lower school levels (Preschool, Primary vs. Upper-Secondary School), as well as project-based learning and gamification. Once again, this reflects the tendency in Upper-Secondary Schools to adopt a teaching approach that is oriented towards disciplinary learning, as in conventional school settings. These findings are again in line with the literature (Benigno et al., 2017).

With regard to technologies, teachers declared more frequent use of digital devices in general; in particular, of tablets and smartphones. These mobile devices played an important role during the emergency phase to facilitate contacts with families and to find resources and activities for hospitalized children. Here, the data show significant differences between school levels, with Secondary School teachers apparently making more use of PCs or laptops.

Overall, the teachers seemed to have acquired greater competence in the use of a variety of technological resources, particularly those they also used during face-to-face teaching. Indeed, they declared greatly increased use after the pandemic (König et al., 2020; Whalen, 2020).

Teachers’ responses regarding the use of technological resources during remote education indicate that the most frequently used were the electronic school roll, videoconferencing systems, instant messaging, and platforms for remote education. This denotes the use of varied resources, ranging from more institutional ones, such as the electronic school roll, to more informal ones like instant messaging. This breadth between formal and informal facilitates direct and immediate communication between teachers and students, which is an important aspect in the context of the SiHo.

As far as differences among school levels, data relating to the use of technological resources during remote education once again highlight the higher frequency of use of videoconferencing, apps, and writing environments by Upper-Secondary School teachers.

Some interesting points emerged from the analysis of the three open-ended questions in the last section of the questionnaire. The mandatory adoption of remote education has led to a significant change both in the acquisition of technological skills and in the need to reorganize and innovate one’s teaching by requiring teachers to plan and use new methodologies. This finding is in line with results from a previous study by Benigno et al. (2020).

Distance teaching was perceived both as a significant means of creating and maintaining contacts with hospitalized students but also as a resource for teachers to use in the organizational management of their teaching activities.

In light of their professional experience, the teachers reported a number of elements in SiHo services that they believe could be improved. For example, they pointed to the need for better technological solutions (both in terms of devices and infrastructures) and resources to support distance learning. They argued that distance learning can help to improve SiHo and can support and increase communication with the mainstream class to which the hospitalized learner belongs.

## Conclusion

This study, which stemmed from two o previous studies on the topic, was chiefly intended to detect whether and how the events and regulations affecting education in Italy during the COVID-19 pandemic also impacted the country’s SiHo services as far as the teaching practices and the use of technologies.

With regard to practices, the results are in line with the previous research conducted in 2017 (Benigno et al., 2017), showing little differentiation in instructional methodologies regardless of whether teaching is conducted face-to-face or remotely. This can either be explained as the result of the specific needs of hospitalized students or in terms of the scarce familiarity most SiHo teachers have with the opportunities that technologies provide and the pedagogical implications of technology integration in the teaching practice. Nevertheless, some of the teachers in this study demonstrated an awareness that the transition to distance education and technology integration in general requires careful planning of learning activities and the adoption of new methodologies.

As to technology integration, the results showed that teachers perceive a significant improvement in their competencies and have positive attitudes towards distance education, which is seen as an important means to support communication with hospitalized students’ mainstream schools. Indeed, SiHo teachers placed special importance on establishing and maintaining contact between hospitalized children and their mainstream school teachers and classmates, a factor that ensures educational continuity and counteracts the social isolation that these students face.

Despite the push for methodological and technological innovation experienced during the COVID emergency, the changes detected remain limited, highlighting the need for investment with respect to the technological improvements (from hub schools but also from hospitals, especially with respect to Internet provision) and for further efforts to ensure methodological innovation to which training interventions seem to be only a building block. Changes in this direction would promote the operation of SiHo teachers and a more adequate response to the needs of hospitalized students.

## Limits of the Study

This study was carried out to complement the findings of a qualitative study conducted in 2020. The need to collect data from a wide number of teachers led us towards a quantitative approach; the (mainly closed) nature of the questions in the questionnaire limited the insight that could be gained from the responses, leaving some issues open. To counterbalance this limit, three open questions were added at the end of the questionnaire, and they proved to be highly valuable in providing informative data.

## Implications for Further Research

Activation of remote connections with students as a result of the pandemic has proven an asset for many schools. This undoubtedly also has implications for the operation of SiHo teachers and brings with it both advantages and challenges. Future studies could analyse the changes resulting from these new set-ups and their impact on teachers and students.

## Additional File

The additional file for this article can be found as follows:

10.5334/cie.65.s1 Supplementary File 1. Appendix: Questionnaire.

## Footnotes

Ciampolini (1993) described this method as “not a univocal methodology, but a set of methodologies – or even simple procedures, techniques, artifices dictated by experience – designed to speed up teaching while respecting scientific rigour” (p. 3).
