# Supplementary material for: Italian Hospital Teachers’ Perceptions of Technological and Methodological Innovations after the COVID-19 Pandemic
Source: Contin Educ. 2023 Apr 14;4(1):67–82. doi: 10.5334/cie.65 (PMC11104358; doi:10.5334/cie.65)
Supplement: Supplementary File 1. — Appendix: Questionnaire. [file cie-4-1-65-s1.pdf]

Appendix

Questionnaire

The School in Hospital After the COVID Pandemic

Dear School in Hospital teacher,

The current school year has required of teachers of all grades and levels a high degree of flexibility to adapt to the constant organisational changes due to the management of the pandemic. In order to understand how the School in Hospital has changed its organisation, we developed this questionnaire, in collaboration between the Istituto per le Tecnologie didattiche del Consiglio Nazionale delle Ricerche (ITD-CNR), the Scuola Polo della Regione Liguria - Istituto Comprensivo di Sturla (Ge) and the Istituto di Istruzione Superiore "E.Montale" (Ge). The main objective is to understand what changes have taken place in hospital teaching and what responses have been implemented.

The questionnaire is divided into six sections:

- the first refers to personal data;
- the second investigates organisational aspects, specifically how hospital teaching was managed between face-to-face and distance instruction and what actions were required;
- the third refers to methodological aspects;
- the fourth section investigates how technologies were used;
- the fifth section investigates the management of distance teaching with the mainstream schools that hospitalised students attend;
- the sixth section stimulates reflection on the effects of the pandemic on the hospital school.

The completion of the survey takes about 15 minutes.

We would be grateful if you could help us disseminate the questionnaire among your colleagues.

We thank you for your valuable cooperation and assure you that the answers you provided by you will only be processed for research purposes and in full compliance with the current law on privacy.

SECTION A: PERSONAL INFORMATION

1. GENDER
    a. Male
    b. Female
    c. I prefer not to answer
2. AGE
    a. between 19 and 29 years old
    b. between 30 and 39 years old
    c. between 40 and 49 years old
    d. over 50 years old
3. EDUCATIONAL BACKGROUND
    a. Diploma
    b. Degree
4. STATE THE NAME OF YOUR SCHOOL
    a. …………………………………………

5. STATE THE NAME OF THE HOSPITAL SCHOOL IN WHICH YOU WORK
    a. …………………………………………..
6. STATE THE HOSPITAL WARD IN WHICH YOU WORK
    a. ……………………………………….
7. CHOOSE THE PROVINCE IN WHICH YOU WORK
    a. ……………………………………….
8. SPECIFY THE SCHOOL LEVEL
    a. Kindergarten
    b. Elementary
    c. Lower Secondary school
    d. Upper Secondary school
9. SPECIFY YOUR TEACHING AREA
    a. Humanities
    b. Scientific
    c. Foreign languages
    d. Technologies
    e. Art
    f. Other
10. HOW MANY YEARS HAVE YOU BEEN TEACHING IN SCHOOL IN HOSPITAL?
    a. Less than two years
    b. Two to five years
    c. Six to ten years
    d. More than ten years

SECTION B: TEACHING ORGANIZATION

11. WHERE DO YOU TEACH (in which ward)?
    a. Day hospital
    b. In one hospital ward
    c. In two or more hospital wards
    d. Other
12. HOW AND HOW FREQUENTLY DO YOU INTERACT WITH HEALTHCARE STAFF? Indicate for all the situations listed above how often they occur (from never to always)

	NEVER	SOMETIMES	WHEN IT IS NECESSARY	OFTEN	ALWAYS
As teacher, I am included and attend meetings with the healthcare staff					
Doctors contact me to share information					
Nurses contact me to share information					
I contact doctors to ask for information and support about my students					
I contact nurses to ask for information and support about my students					
Other					

13. EXPLAIN WHAT YOU MEAN WITH 'OTHER':
………
………
……
14. WHERE THERE ANY CHANGES, AFTER THE OUTBREAK OF THE COVID-19 PANDEMIC, IN YOUR RELATIONS WITH THE HEALTHCARE STAFF?
    a. Yes
    b. No
    c. I don't have the data to answer
15. IF YES, PLEASE INDICATE WHAT CHANGES YOU HAVE FOUND:
………
………
……
16. DURING THIS SCHOOL YEAR IN WHICH MANNER DID YOU CARRY OUT YOUR TEACHING ACTIVITIES IN SCHOOL HOSPITAL:
For each of the two options, indicate how often they have occurred (from never to always).

	NEVER	FOR FEW MONTHS	FOR ABOUT HALF OF THE YEAR	FOR MOST OF THE TIME	ALWAYS
Face-to-face					
Distance					

17. PLEASE INDICATE WHETHER, AND WHAT CHANGES, HAVE OCCURRED IN TERMS OF SPACES AND TIMING IN FACE-TO-FACE TEACHING ACTIVITIES:
To answer the question, it is necessary to select at least one of the answers. Multiple answers can be chosen. It is possible to add the description of the chosen option on the right field. In case there were no changes you can select the answer "no change" and continue with the questionnaire.

    a. Spaces
    b. Time
    c. No change
    d. I don't have the data to answer

18. HOW DID YOU MANAGE TO INITIATE CONTACTS WITH STUDENTS FOR REMOTE EDUCATION (DURING LOCKDOWN)?

	NEVER	FEW CASES	OFTEN	ALWAYS
I was already in contact before remote education started				
Through posters/flyers in the wards				
Through the efforts of healthcare staff				
By promoting the service on the hospital website				
Other				

19. EXPLAIN WHAT YOU MEAN WITH 'OTHER':
………
………
……

SECTION C: METHODOLOGY

20. INDICATE HOW OFTEN YOU HAVE USED THE FOLLOWING TEACHING STRATEGIES SINCE THE BEGINNING OF THE PANDEMIC FOR FACE-TO-FACE TEACHING:

	NEVER	RARELY	SOMETIMES	OFTEN	ALWAYS
Lectures					
Group work					
Brainstorming					
Drill & practice					
Project-based learning					
Gamification					
Playful activities					
Teaching essential knowledge					
Other strategies					

21. EXPLAIN WHAT YOU MEAN WITH 'OTHER STRATEGIES' FOR FACE-TO-FACE TEACHING:
……
……
……

22. INDICATE HOW OFTEN YOU HAVE USED THE FOLLOWING TEACHING STRATEGIES SINCE THE BEGINNING OF THE PANDEMIC FOR DISTANCE LEARNING:

	NEVER	RARELY	SOMETIMES	OFTEN	ALWAYS
Lectures					
Group work					
Brainstorming					
Drill & practice					
Project-based learning					
Gamification					
Playful activities					
Teaching essential knowledge					
Other strategies					

23. EXPLAIN WHAT YOU MEAN WITH 'OTHER STRATEGIES' FOR DISTANCE LEARNING:
……
……
……

SECTION D: TECHNOLOGY

24. PLEASE INDICATE WHETHER YOU USED THE FOLLOWING TOOLS DURING YOUR TEACHING (BOTH DURING DESIGNING AND PERFORMING PHASE):

	USED	NOT USED
PC/Laptop		
Tablet		
Smartphone		

25. PLEASE INDICATE WHETHER THE USE OF THE FOLLOWING TOOLS HAS CHANGED SINCE THE PANDEMIC IN TERMS OF FREQUENCY:

	INCREASED DURING PANDEMIC	UNCHANGED DURING PANDEMIC	DECREASED DURING PANDEMIC	I DON'T HAVE THE DATA TO ANSWER
PC/Laptop				

Tablet				
Smartphone				

26. PLEASE INDICATE WHETHER YOU USED THE FOLLOWING APPLICATIONS AND DIGITAL ENVIRONMENTS DURING FACE-TO-FACE TEACHING:

	USED	NOT USED
One or more common productivity applications (word processing, spreadsheets, presentations, etc.)		
Shared online workspaces (Google Drive, Dropbox, etc.)		
Collaborative writing applications (Wiki, Google Docs, Book creator, etc.)		
Educational robotics and coding applications (Bee bot, Ozobot, Scratch, etc.)		
Other cloud applications (Padlet, Learning Apps, Kahoot, etc.)		
Online educational resources (Lessons and teaching materials available online)		

27. PLEASE INDICATE WHETHER THE USE OF THE FOLLOWING APPLICATIONS AND DIGITAL ENVIRONMENTS HAS CHANGED DURING THE PANDEMIC:

	INCREASED DURING PANDEMIC	UNCHANGED DURING PANDEMIC	DECREASE DURING PANDEMIC	I DON'T HAVE THE DATA TO ANSWER
One or more common productivity applications (word processing, spreadsheets, presentations, etc.)				
Shared online workspaces (Google Drive, Dropbox, etc.)				
Collaborative writing applications (Wiki, Google Docs, Book creator, etc.)				
Educational robotics and coding applications (Bee bot, Ozobot, Scratch, etc.)				
Other cloud applications (Padlet, Learning Apps, Kahoot, etc.)				
Online educational resources (Lessons and teaching materials available online)				

28. WHICH OF THE FOLLOWING TOOLS DID YOU USE WITH THE HOSPITALIZED STUDENTS DURING DISTANCE LESSONS?

	NEVER	RARELY	SOMETIMES	OFTEN	ALWAYS
Electronic school roll					
Videoconferencing systems (Skype, Zoom, Meeting, etc.)					
Instant Messaging (WhatsApp, Telegram, etc.)					
Platforms for remote education (Moodle, Classroom, Microsoft Teams, etc.)					
Publishers’ applications (Hub Scuola,					

Dea Link, Bsmart, etc.)					
Shared online workspaces (Google Drive, Dropbox, etc.)					
Collaborative writing applications (Wiki, Google Docs, Book creator, etc.)					
Other cloud applications (Padlet, Learning Apps, Kahoot, etc.)					
Online educational resources (Lessons and teaching materials available online)					

SECTION E: HOSPITALIZED STUDENTS AND REMOTE EDUCATION WITH MAINSTREAM SCHOOLS

29. WERE THERE HOSPITALIZED STUDENTS WHO DID NOT BENEFIT FROM THE SCHOOL IN HOSPITAL BECAUSE THEY WERE ALREADY ATTENDING DISTANCE EDUCATION WITH THE MAINSTREAM SCHOOL??
    a. Yes
    b. No
30. WHAT DO YOU THINK ARE THE REASONS BEHIND THIS CHOICE?

    ………………………………….

31. PLEASE INDICATE HOW MANY STUDENTS YOU TUTORED (DIVIDED INTO SHORT, MEDIUM AND LONG STAYS), WHO HAD BENEFITED FROM THE REMOTE EDUCATION PROVIDED BY THEIR MAINSTREAM SCHOOL SINCE THE BEGINNING OF THE PANDEMIC

	ALL OF THEM	MOST OF THEM	A SMALL PART OF THEM	NONE OF THEM	N/A
Short hospital stay					
Medium hospital stay					
Long hospital stay					

32. PLEASE INDICATE HOW MANY STUDENTS JOIN THEIR MAINSTREAM SCHOOLS AT A DISTANCE FOR EACH OF THE MODALITIES LISTED BELOW:

	ALL OF THEM	MOST OF THEM	A SMALL PART OF THEM	NONE OF THEM
They worked independently				
They needed hospital teacher’s support to keep up and/or integrate parts of the lessons attended				
They needed hospital teacher’s support in all lessons they attended				

33. PLEASE INDICATE HOW MANY STUDENTS WHO HAVE BEEN ABLE TO ATTEND CLASSES OF THEIR MAINSTREAM SCHOOL CLASS AT DISTANCE, ACCORDING TO THE MODES LISTED BELOW:

	ALL OF THEM	MOST OF THEM	A SMALL PART OF THEM	NONE OF THEM	N/A
Every day					
A few hours per week					
For specific activity					
On an unscheduled basis					

34. HAVE THE STUDENTS YOU TUTORED GOT IN TOUCH WITH THEIR CLASSMATES SINCE THE BEGINNING OF THE PANDEMIC?

	ALL OF THEM	MOST OF THEM	A SMALL PART OF THEM	NONE OF THEM	N/A
For social and friendship purposes					
For educational purposes					

35. HAVE THE STUDENTS YOU TUTORED GOT IN TOUCH WITH THEIR MAINSTREAM SCHOOL TEACHERS SINCE THE BEGINNING OF THE PANDEMIC?

	ALL OF THEM	MOST OF THEM	A SMALL PART OF THEM	NONE OF THEM	N/A
For reasons not related to school					
For reasons related to school and educational activities					

36. WITH RESPECT TO THE DIFFERENT TYPES OF CONTACT WITH THE MAINSTREAN SCHOOLS OF YOUR HOSPITALIZED STUDENTS, PLEASE INDICATE ANY CHANGES OBSERVED SINCE THE START OF THE PANDEMIC:

	PRESENT AS IN THE PAST	ABSENT AS IN THE PAST	INCREASED COMPARED TO THE PAST	DECREASED COMPARED TO THE PAST	I DON'T HAVE THE DATA TO ANSWER
Formal communication between you and the mainstream school teachers					
Informal communication between you and the mainstream school teachers					
Exchanging teaching materials with the mainstream school teachers					
Planning activities with the student's class to be carried out asynchronously (without direct contact between the hospitalized student and their class)					
Planning activities with the student's class to be carried out in synchronous mode (with direct					

contact between the hospitalized student and their class)					
Possibility of obtaining information on the student's educational pathway before hospitalization					

37. DID YOU NOTICE ANY OTHER CHANGES, IN ADDITION TO THOSE MENTIONED IN THE PREVIOUS QUESTION? IF YES, PLEASE DESCRIBE WHICH ONES:
……
……
………

REFLECTION ON THE PANDEMIC'S EFFECTS ON SCHOOL IN HOSPITAL

38. WHAT POSITIVE CHANGES IN SCHOOL IN HOSPITAL HAVE EMERGED IN YOUR EXPERIENCE DURING THE PANDEMIC?
……
……
……

39. WHAT CRITICALITIES IN SCHOOL IN HOSPITAL HAVE EMERGED IN YOUR EXPERIENCE DURING THE PANDEMIC?
……
……
……

40. IN THE LIGHT OF YOUR EXPERIENCE DURING THE PANDEMIC, WHAT ASPECTS OF SCHOOL IN HOSPITAL CAN BE IMPROVED AND FOR WHAT PURPOSE?
……
……
……
